# Quantum Control Design by Lyapunov Trajectory Tracking and Optimal Control

**DOI:** 10.3390/e26110978

**Published:** 2024-11-15

**Authors:** Hongli Yang, Guohui Yu, Ivan Ganchev Ivanov

**Affiliations:** 1 College of Big Data, Qingdao Huanghai University, Qingdao 266427, China; 2 College of Mathematics and Systems Science, Shandong University of Science and Technology, Qingdao 266590, China; 202282150047@sdust.edu.cn; 3 Faculty of Economics and Business Administration, St. Kl. Ohridski Sofia University, 1113 Sofia, Bulgaria

**Keywords:** quantum system, Lyapunov function, optimal control, spin-1/2 particle system

## Abstract

In this paper, we investigate a Lyapunov trajectory tracking design method that incorporates a Schrödinger equation with a dipole subterm and polarizability. Our findings suggest that the proposed control law can overcome the limitations of certain existing control laws that do not converge. By integrating a quadratic performance index, we introduce an optimal control law, which we subsequently analyze for stability and optimality. We also simulate the spin-1/2 particle system to illustrate our results. These findings are further validated through numerical illustrations involving a 3D, 5D system, and a spin-1/2 particle system.

## 1. Introduction

The quantum theory stands as one of the most remarkable accomplishments of humanity in the 20th century. It unveils the structure, properties, and laws of motion governing matter on a microscopic scale, ushering our understanding from the realm of the macroscopic to that of the microscopic domain. A sequence of renowned experiments conducted from the late 19th century through to the 1930s—including investigations into blackbody radiation [1,2], the photoelectric effect [3,4], Compton scattering [5,6], and Young’s double-slit interference with electrons—have collectively demonstrated that wave–particle duality [7] is a pervasive characteristic of microscopic matter. This has led to the discovery of phenomena distinct from classical systems, such as quantum coherence [8] and quantum entanglement. Concurrently, there has been a burgeoning interest in leveraging quantum theory and methodologies across diverse disciplines—ranging from the fission of molecular bonds during chemical reactions [9] to alterations in laser intensity within physics. The swift progression of quantum communication and quantum computing underscores the paramount importance of managing quantum states adeptly.

The concept of quantum control originated in 1938, and the introduction of laser techniques for controlling quantum coherent states led to a rapid advancement in this field during the 1970s. This area of study focuses on manipulating systems to reach specific target states. Optimal control involves modifying the external control fields to either maximize or minimize the current expected value, which is a fundamental issue in quantum control research. Quantum optimal control garnered significant interest from scientists in the late 1980s. In 1988, Rabitz et al. [10] provided an in-depth explanation of the optimal control challenges in quantum systems, establishing a foundational framework for future studies in quantum optimal control. Following their work, Rabitz and colleagues expanded the application of quantum optimal control theory into other domains. More recently, D’Alessandro et al. [11] have primarily investigated two-level quantum systems, achieving notable results in optimal quantum control through the method of Lie group decomposition analysis [12,13,14]. The Lyapunov function method is an effective method for quantum control systems described by the Schrodinger equation [15]; more references about the Lyapunov function method in quantum control can be found in [15,16,17,18].

Quantum systems are generally categorized into two primary classes: closed quantum systems and open quantum systems. This paper concentrates on a closed quantum system, which can be described and manipulated using either the Schrödinger equation or the non-dissipative Liouville von Neumann equation.

In this paper, we consider the evolution of a quantum system with a wave function ψ(t) under the external influence of a laser field that satisfies the time-dependent Schrödinger equation (TDSE).

(1) $$i|\dot{\psi (t)}\rangle =H\left(t\right)|\psi \left(t\right)\rangle ,$$

where H(t) is the Hermitian operator, and the control is achieved by selecting the appropriate laser intensity u(t). When the laser is turned off, H(t) is the Hamiltonian amount inside the system, expressed in $H_{0}$. When the laser is turned on, H(t) is the sum of $H_{0}$ and other additions to the system’s interaction with the laser field. Here, we consider the Schrödinger equation with dipole subterm and polarizability coupling, both of which can be represented by first-order terms [19], so that the Hamiltonian can be expressed as $H\left(t\right)=H_{0}+u_{1}\left(t\right)H_{1}+u_{2}\left(t\right)H_{2}$.

The primary objective of this study is to identify an appropriate control field for the Hamiltonian $H\left(t\right)=H_{0}+u_{1}\left(t\right)H_{1}+u_{2}\left(t\right)H_{2}$ by monitoring the control process through adaptive feedback $u_{i}\left(t\right)\left(i=1,2\right)$, where $H_{0},H_{1},$ and $H_{2}$ are Hermitian matrices with complex coefficients and the control field is laser intensity $u_{i}\left(t\right)\in R$, which belongs to the set of real numbers R.

The controllability of finite-dimensional quantum systems is governed by specific equations

(2) $$i|\dot{\psi (t)}\rangle =\left(H_{0}+u_{1}\left(t\right)H_{1}+u_{2}\left(t\right)H_{2}\right)|\psi \left(t\right)\rangle .$$

This paper delves into the realm of optimal control [20,21,22], drawing from the extensive literature on Lyapunov functions [23,24,25,26,27,28]. The stability theory pioneered by Lyapunov at the end of the 19th century is not limited to univariate, linear, and constant systems but also extends to multivariate, nonlinear, and time-varying systems. Lyapunov introduced two methods to tackle the stability issue: the indirect method and the direct method. The indirect method assesses motion stability by solving the differential equation; that is, the stability of the original nonlinear system is determined by analyzing the eigenvalue distribution of the nonlinear system’s linearization equation. Conversely, Lyapunov’s direct method is more qualitative, eliminating the need to solve nonlinear differential equations. Instead, it involves constructing a Lyapunov function and examining its positive definiteness and the negative or semi-negative definiteness of its derivative with respect to time to derive a stable conclusion. The concept of quantum Lyapunov control entails constructing a Lyapunov function to design the corresponding control law. However, the method presented in this study can offer valuable insights into state space and serve as an initial step toward more practical designs, incorporating realistic measurements and feedback.

In principle, as long as a quantum system is controllable, one can design the required control laws. The control law designed using Lyapunov’s method does not diverge from a closed quantum system. Different Lyapunov functions have different control laws designed, and it is very important to choose the appropriate Lyapunov function to make the algorithm simple. The contribution of this paper is that we explore a quantum control design strategy that utilizes the Lyapunov function and the optimal control law for a quantum control system. We propose two distinct control law design methods. The results demonstrate that the proposed control laws are effective. Our control law is more straightforward than the one presented in [19].

The structure of this paper is as follows: In Section 2, we present the primary symbolic and Lyapunov-based control laws devised for a specific scenario, along with numerical simulations conducted on both a 3D and a 5D test system. In Section 3, we extend our study to a spin-1/2 particle system, applying the control methods outlined in Section 2. By integrating a quadratic performance index, we formulate an optimal control strategy, examine its stability and optimality properties, and conduct further simulations on the spin-1/2 particle system. Finally, we summarize our key findings in Section 4.

## 2. Control Law Design

### 2.1. Quantum System Described by Schrödinger Equation (TDSE)

For simplicity, we add a second control ω [29]. Therefore, we consider the following control system:

(3) $$i|\dot{\psi (t)}\rangle =\left(H_{0}+u_{1}\left(t\right)H_{1}+u_{2}\left(t\right)H_{2}+\omega \left(t\right)I\right)|\psi \left(t\right)\rangle .$$

Among them, ω∈R is a new control, which is the role of adjusting the degrees of freedom. We can arbitrarily choose it, assuming that the control $u_{i}$ and ω given by (3) are independent of each other.

**Remark** **1.** *The wave function $\psi ={\left(\psi_{i}\right)}_{i=1}^{n}$ is a vector on $C^{n}$, proving that $\sum_{i=1}^{n}{\left|\psi_{i}\right|}^{2}=1$. It exists on the unit sphere of $C^{n}$. Physically, for any global phase t→θ(t)∈R, the probability amplitudes ψ and $e^{i\theta (t)}\psi$ describe the same physical state. This point has important implications for the geometry of the physical state space; when θ∈R, there is $\psi_{1}=exp\left(i\theta \right)\psi_{2}$, and two probability amplitudes $\psi_{1}$ and $\psi_{2}$ can be determined. Considering this non-trivial geometry, we add a second control law ω corresponding to $\dot{\theta }$.*

### 2.2. Lyapunov Control Design

The objective of this article is to transition the system to the final state $|\psi_{f}\rangle$, and to achieve this, we construct an appropriate state feedback. The asymptotic stability of the control system is guaranteed by minimizing the distance between the actual state and the final state. Although there are various methods to calculate the distance between two states, employing the Hilbert–Schmidt distance simplifies this computation significantly.

Here, we choose the function based on the Hilbert–Schmidt distance between the controlled state |ψ〉 and the desired target $|\psi_{f}\rangle$ as the Lyapunov function [30], i.e., $1-|\langle \psi_{f}|\psi \rangle {|}^{2}$. The Hilbert–Schmidt distance measures the “closeness” of two quantum states in terms of their inner product. This choice of the Lyapunov function is motivated by the fact that it is zero when the system reaches the target state and positive otherwise, making it suitable for stability analysis.

(4) $$V=\frac{1}{2}(1-|\langle \psi_{f}|\psi \rangle {|}^{2}),$$

where $|\langle \psi_{f}|\psi \rangle {|}^{2}$ represents the transition probability from |ψ〉 to $|\psi_{f}\rangle$.

Considering the control system of Equation (3), in quantum control, the target state is usually the eigenstate of the Hamiltonian $H_{0}$, i.e., the target state $|\psi_{f}\rangle$ satisfies the following conditions:

(5) $$H_{0}|\psi_{f}\rangle =\lambda_{f}|\psi_{f}\rangle .$$

The first-order time derivative of a *V* can be calculated:

(6) $$\begin{array}{c} \begin{array}{cc} \dot{V} & =-\langle \psi_{f}|\psi \rangle \langle \psi_{f}|\dot{\psi }\rangle \\ \; & =-u_{1}Im\langle \psi |\psi_{f}\rangle \langle \psi_{f}|H_{1}|\psi \rangle -u_{2}Im\langle \psi |\psi_{f}\rangle \langle \psi_{f}|H_{2}|\psi \rangle -\omega Im\langle \psi |\psi_{f}\rangle \langle \psi_{f}|\psi \rangle , \end{array} \end{array}$$

where Im represents the imaginary part.

For convenience, let $I_{1}=Im\langle \psi |\psi_{f}\rangle \langle \psi_{f}|H_{1}|\psi \rangle$, $I_{2}=Im\langle \psi |\psi_{f}\rangle \langle \psi_{f}|H_{2}|\psi \rangle$, and $I_{3}=$$Im\langle \psi |\psi_{f}\rangle \langle \psi_{f}|\psi \rangle$, and then Equation (6) can be abbreviated as

$$\dot{V}=-u_{1}I_{1}-u_{2}I_{2}-\omega I_{3}.$$

In this case, to find the control $u_{i}$ and ω, such that $\dot{V}$ is less than or equal to 0, we can set $u_{1}I_{1}$, $u_{2}I_{2}$ and $\omega I_{3}$ to be greater than or equal to 0, i.e., take $u_{i}$ as an example. The function form $f_{i}\left(•\right)$ of $u_{i}$ satisfies $f_{i}\left(I_{i}\right)I_{i}\leq 0$. Obviously, when the image of function $y_{i}=f_{i}\left(I_{i}\right)$ passes the origin of plane $I_{i}-y_{i}$ monotonically and lies in quadrant II or IV, the above requirement will be satisfied. At the same time, $f_{i}\left(I_{i}\right)I_{i}=0$ iff $I_{i}=0$.

For example, let [16]

(7) $$\begin{array}{c} \left\{\begin{array}{c} u_{i}=\frac{I_{i}}{{\left(1+\frac{1}{\alpha_{i}}I_{i}\right)}^{2}}\\ \omega =I_{3} \end{array}\right. \end{array}\left(i=1,2\right),$$

where $\alpha_{i}$ is a strictly positive parameter, so the following is obtained:

$$\dot{V}=-\left(\sum_{i=1}^{2}\left(\frac{I_{i}}{{\left(1+\frac{1}{\alpha_{i}}I_{i}\right)}^{2}}\right)I_{i}\right)-I_{3}^{2},$$

$$\dot{V}=-\left(\sum_{i=1}^{2}\frac{I_{i}^{2}}{{\left(1+\frac{1}{\alpha_{i}}I_{i}\right)}^{2}}\right)-I_{3}^{2}\leq 0.$$

Therefore, *V* is decreasing.

### 2.3. Examples and Simulations

We use the control law (7) to perform numerical simulations in a 3D system first.

**Example** **1.** *Numerical simulation of a 3D test system with $H_{0},H_{1},$ and $H_{2}$, where $H_{0},\;H_{1},$ and $H_{2}$ are*

(8) $$H_{0}=\left[\begin{array}{ccc} 0 & 0 & 0\\ 0 & 1 & 0\\ 0 & 0 & 1 \end{array}\right],\;H_{1}=\left[\begin{array}{ccc} 1 & 0 & 0\\ 0 & 1 & 1\\ 0 & 1 & 1 \end{array}\right],\;H_{2}=\left[\begin{array}{ccc} 0 & 1 & 1\\ 1 & 0 & 1\\ 1 & 1 & 0 \end{array}\right].$$

**Figure 1:**
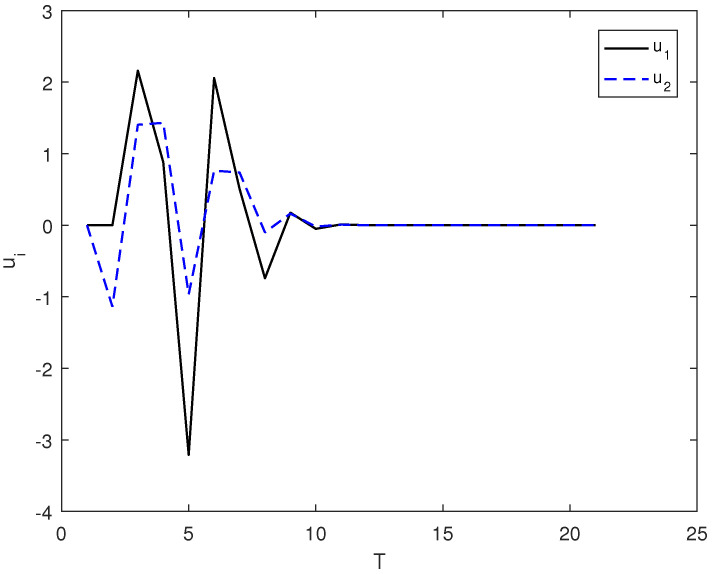
The evolution of the control law $u_{i}$ adopts the control law of (7) under the system of (8).

**Figure 2:**
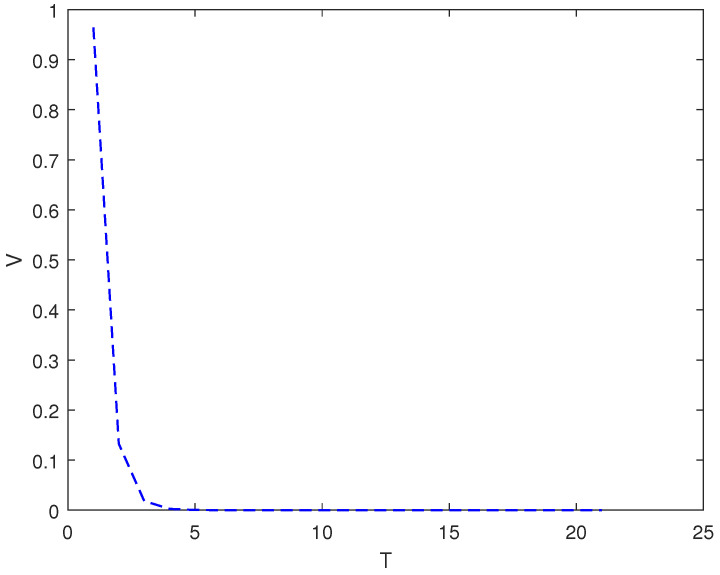
The evolution of the Lyapunov function *V* with time under system (8) and control law (7).

*In this example, the initial state is $|\psi \left(0\right)\rangle ={\left(0,0,1\right)}^{T}$ and the target state is $|\psi_{f}{\rangle =\left(1,0,0\right)}^{T}$, using the control law of Equation (7), $\alpha_{1}=9$, $\alpha_{2}=6$, and the total length of time is T. Figure 1 shows the evolution of the control law $u_{i}$ corresponding to system (8). Figure 2 shows the evolution of the Lyapunov function V with time T.*

**Example** **2.** *Consider another 3D test system with $H_{0},H_{1}$, and $H_{2}$, where $H_{0},\;H_{1}$, and $H_{2}$ are*

(9) $$H_{0}=\left[\begin{array}{ccc} 0 & 0 & 0\\ 0 & 1 & 0\\ 0 & 0 & \frac{3}{2} \end{array}\right],\;H_{1}=\left[\begin{array}{ccc} 0 & 1 & 0\\ 1 & 0 & 0\\ 0 & 0 & 0 \end{array}\right],\;H_{2}=\left[\begin{array}{ccc} 0 & 0 & 1\\ 0 & 0 & 0\\ 1 & 0 & 0 \end{array}\right].$$

**Figure 3:**
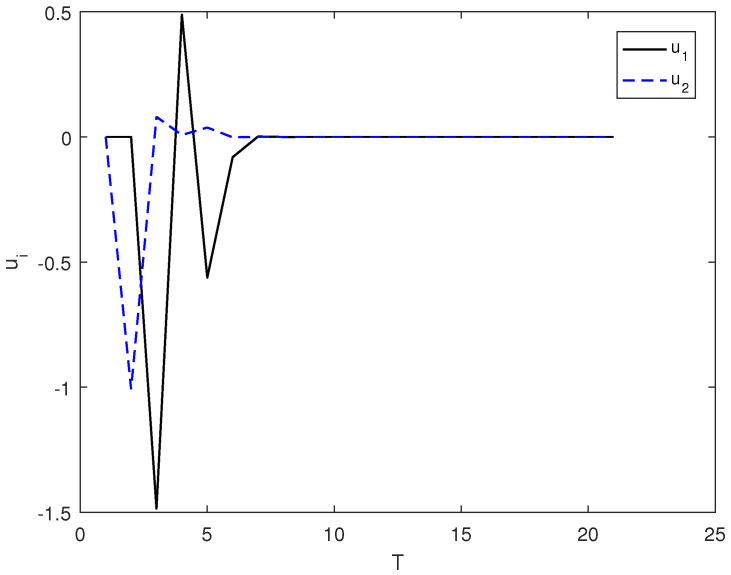
The evolution of the control law $u_{i}$ adopts the control law of (7) under the system of (9).

**Figure 4:**
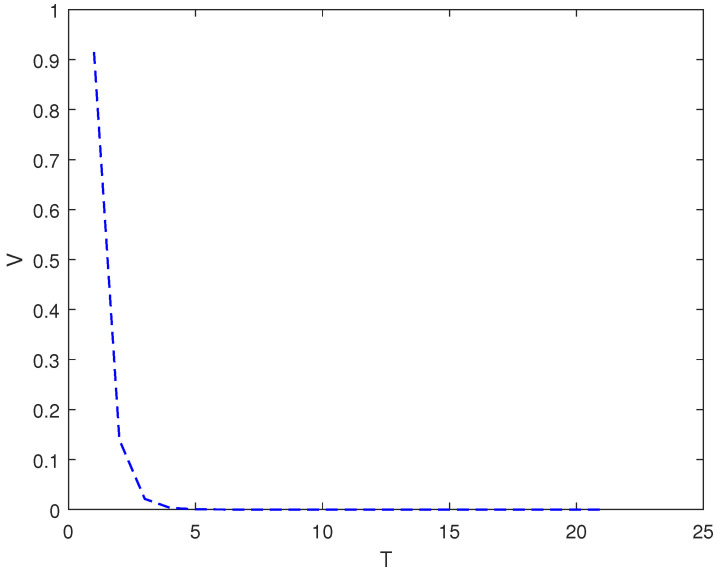
The evolution of the Lyapunov function *V* with time under system (9) and control law (7).

*In this example, the initial state is $|\psi \left(0\right)\rangle ={\left(0,0,1\right)}^{T}$ and the target state is $|\psi_{f}{\rangle =\left(1,0,0\right)}^{T}$; using the control law of Equation (7), $\alpha_{1}=2$, $\alpha_{2}=\frac{1}{2}$, and the total length of time is T. Figure 3 shows the evolution of the control law $u_{i}$ corresponding to system (9). Figure 4 shows the evolution of the Lyapunov function V with time T.*

**Remark** **2.** *In this example, the proposed control law makes system converge, but the control law in [19] cannot make the system convergent.*

To determine the applicability of the control law given in Equation (7) within a five-dimensional system, we need to analyze both the structure and dimensionality of the control law and the system.

**Example** **3.** *Numerical simulation of a 5D test system with $H_{0},H_{1},$ and $H_{2}$, where $H_{0},H_{1},$ and $H_{2}$ are*

(10) $$\begin{array}{c} H_{0}=\left[\begin{array}{ccccc} \frac{1}{2} & 0 & 0 & 0 & 0\\ 0 & 0 & 0 & 0 & 0\\ 0 & 0 & 1 & 0 & 0\\ 0 & 0 & 0 & 1 & 0\\ 0 & 0 & 0 & 0 & 0 \end{array}\right] \end{array},\;H_{1}=\left[\begin{array}{ccccc} 0 & 0 & 0 & 0 & \frac{1}{5}\\ 0 & 0 & 0 & 0 & \frac{1}{5}\\ 0 & 0 & 1 & 0 & 0\\ 0 & 0 & 0 & 1 & 0\\ \frac{1}{5} & \frac{1}{5} & 0 & 0 & 1 \end{array}\right],\;H_{2}=\left[\begin{array}{ccccc} 0 & \frac{1}{3} & 0 & 0 & 1\\ \frac{1}{3} & 0 & 0 & 0 & 0\\ 0 & 0 & \frac{1}{3} & 0 & 0\\ 0 & 0 & 0 & \frac{2}{3} & 0\\ 1 & 0 & 0 & 0 & \frac{1}{5} \end{array}\right].$$

**Figure 5:**
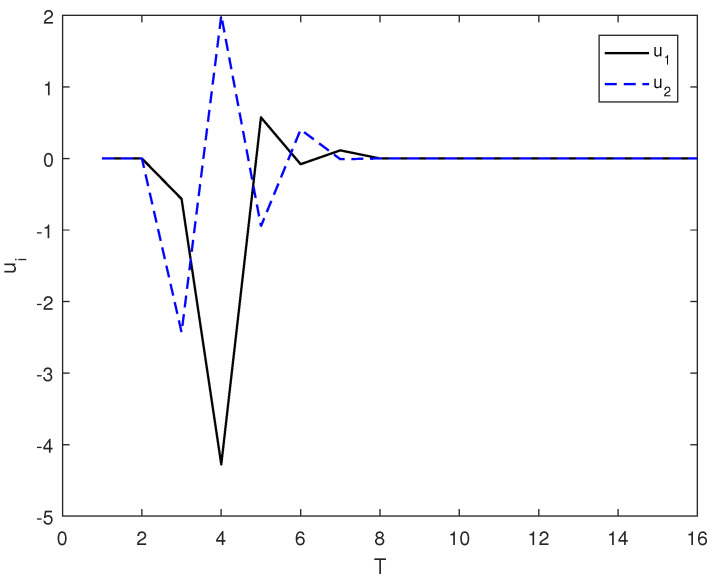
The evolution of the control law $u_{i}$ adopts the control law of (7) under the system of (10).

**Figure 6:**
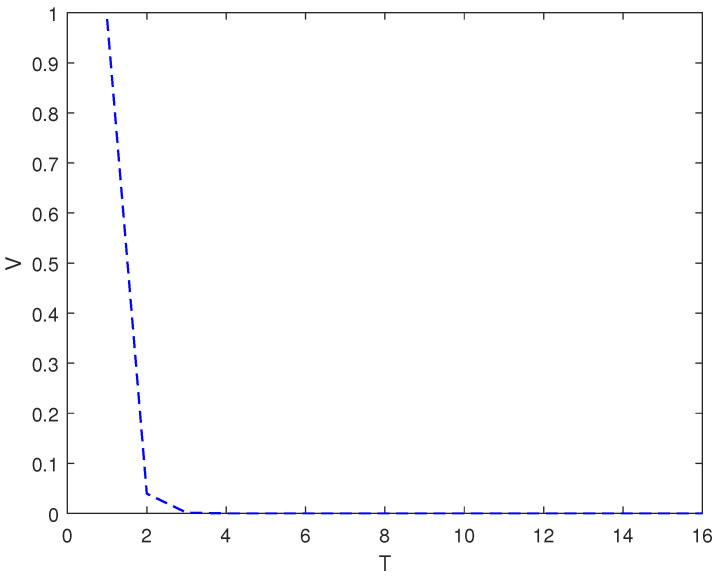
The evolution of the Lyapunov function *V* with time under system (10) and control law (7).

*In this example, the initial state is $|\psi \left(0\right)\rangle ={\left(0,0,0,0,1\right)}^{T}$ and the target state is $|\psi_{f}{\rangle =\left(1,0,0,0,0\right)}^{T}$; using the control law of Equation (7), $\alpha_{1}=6$, $\alpha_{2}=8$, and the total length of time is T. Figure 5 shows the evolution of the control law $u_{i}$ corresponding to system (10). Figure 6 shows the evolution of the Lyapunov function V with time T.*

As it can be seen from Figure 2, Figure 4 and Figure 6, whether it is a 3D system or a 5D system, the control law of Equation (7) can make the initial state of the system asymptotically approach the target state, which is desirable.

## 3. A Spin-1/2 Particle System

### 3.1. Simulation Experiment

Consider a spin-1/2 particle system is controlled by two control fields $u_{1}\left(t\right)$ and $u_{2}\left(t\right)$, and the control field changes the electromagnetic field in the *x* and *y* directions. Spin is discussed in *z*-notation, and the Schrödinger equation corresponding to its wave function can be expressed by Equation (3), where

(11) $$H_{0}=\sigma_{z}=\left[\begin{array}{cc} 1 & 0\\ 0 & -1 \end{array}\right],\;H_{1}=\sigma_{x}=\left[\begin{array}{cc} 0 & 1\\ 1 & 0 \end{array}\right],\;H_{2}=\sigma_{y}=\left[\begin{array}{cc} 0 & -i\\ i & 0 \end{array}\right]$$.

According to the principle of linear superposition, to perform the simplest logical NOT gate, the state |ψ(t)〉 must be transformed between two eigenstates $|0\rangle ={\left[1,0\right]}^{T}$ and $|1\rangle ={\left[0,1\right]}^{T}$. Now let the initial state |ψ(0)〉=|0〉, and the target state $|\psi_{f}\rangle =|1\rangle$.

In this example, in order to make the controlled state |ψ(t)〉 reach the target state $|\psi_{f}\rangle =|1\rangle$, we use the Lyapunov control of formula (7) with $\alpha_{1}=\alpha_{2}=4$, and the total length of time is T.

**Figure 7:**
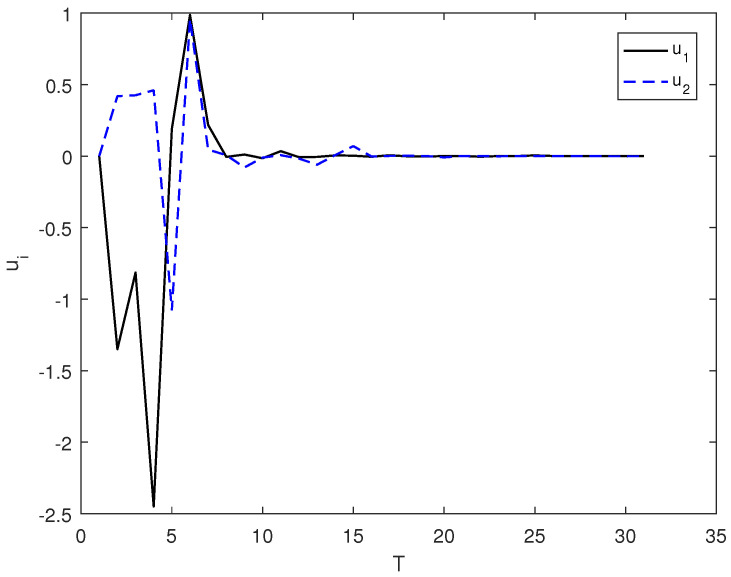
The evolution of the control law $u_{i}$ adopts the control law of (7) under the system of (11).

**Figure 8:**
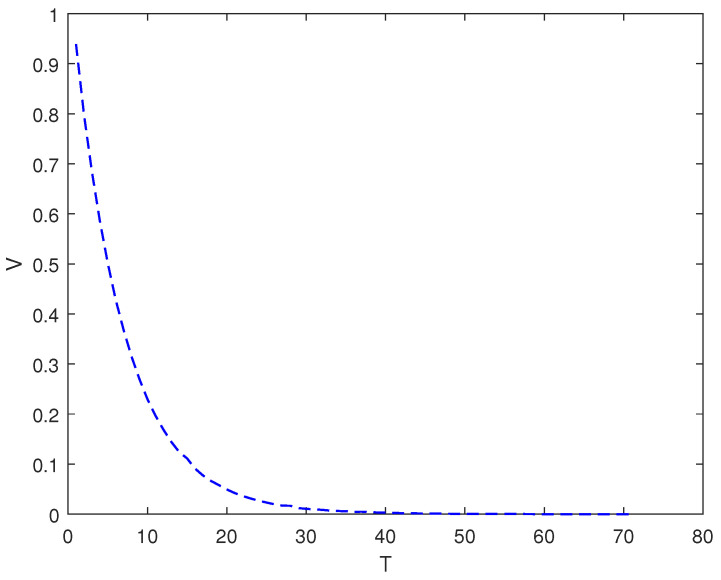
The evolution of the Lyapunov function *V* with time under system (11) and control law (7).

Figure 7 illustrates the evolution of the control law $u_{i}$ under system (11). Figure 8 shows the evolution of the Lyapunov function *V* with time *T*.

### 3.2. Optimal Control

#### 3.2.1. Selection of Optimal Control Law

For system (3), assuming that the target state $|\psi_{f}\rangle$ satisfies condition (5), take $\omega =-\lambda_{f}$, and then $\left(H_{0}+\omega I\right)|\psi_{f}\rangle =0$. The control law is designed by the optimal stable control method proposed by Benallou [31] et al.; choose the real number space to describe system (3), and it is necessary to separate the coefficient matrix of (3) and the real and imaginary parts of the state [32]. Let the controlled state $|\psi \rangle ={\left[x_{1}+ix_{n+1},x_{2}+ix_{n+2}\cdots x_{n}+ix_{2n}\right]}^{T}$, and from the equality of the real and imaginary parts on both sides of (3), we obtain the following state-space equation for the real vector *x*

(12) $$\dot{x}=\left(A+\sum_{i=1}^{m}B_{i}u_{i}\right)x,$$

where $A=\left[\begin{array}{cc} Im(H_{0}+\omega I) & Re(H_{0}+\omega I)\\ -Re(H_{0}+\omega I) & Im(H_{0}+\omega I) \end{array}\right]$, $B_{i}=\left[\begin{array}{cc} Im(H_{i}) & Re(H_{i})\\ -Re(H_{i}) & Im(H_{i}) \end{array}\right]$, Im represents the imaginary part, Re represents the real part, *A* and $B_{i}$ must satisfy $A+A^{T}=0$ and $B_{i}+B_{i}^{T}=0$, and from $\left(H_{0}+\omega I\right)|\psi_{f}\rangle =0$, we can get $Ax_{f}=0$. At this point, the dimension of the real state space becomes 2*n*. The control law of Equation (12) is given by Theorem 1 [33] below.

**Theorem** **1.** *For the state-space equation of system (12), the following performance metrics are given:*

(13) $$J=\frac{1}{2}\int_{0}^{\infty }\left(x^{T}Qx+\sum_{i=1}^{m}\frac{1}{r_{i}}{\left[{\left(x-x_{f}\right)}^{T}PB_{i}x\right]}^{2}+u^{T}Ru\right)dt,$$

*where $u={\left[u_{1},u_{2}\cdots u_{m}\right]}^{T}$, R is a diagonally definite matrix whose elements are greater than 0, that is, $r_{i}>0\left(i=1,2\cdots m\right)$, P is a positively definite symmetric matrix, and Q is a semi-positive definite symmetric matrix that satisfies the equation*

(14) $$PA+A^{T}P=-Q.$$

*Then, there is an optimal control law:*

(15) $$u_{i}^{*}=-\frac{1}{r_{i}}{\left(x-x_{f}\right)}^{T}PB_{i}x\left(i=1,2\cdots m\right),$$

*which guarantees that system (12) is stable and can minimize the performance index (13).*

**Proof** **of Theorem 1.** (i) Stability

Select the following Lyapunov function:

$$V\left(x\right)=\frac{1}{2}{\left(x-x_{f}\right)}^{T}P\left(x-x_{f}\right).$$

Find the first derivative

(16) $$\begin{array}{cc} \dot{V}\left(x\right) & ={\left(x-x_{f}\right)}^{T}P\dot{x}\\ & ={\left(x-x_{f}\right)}^{T}P\left(A+\sum_{i=1}^{m}B_{i}u_{i}\left(t\right)\right)x\\ & ={\left(x-x_{f}\right)}^{T}PAx+\sum_{i=1}^{m}{\left(x-x_{f}\right)}^{T}PB_{i}u_{i}\left(t\right)x\\ & =x^{T}PAx-x_{f}^{T}PAx+\sum_{i=1}^{m}{\left(x-x_{f}\right)}^{T}PB_{i}u_{i}\left(t\right)x\\ & =\frac{1}{2}x^{T}\left(PA+A^{T}P\right)x-x_{f}^{T}PAx+\sum_{i=1}^{m}{\left(x-x_{f}\right)}^{T}PB_{i}u_{i}\left(t\right)x. \end{array}$$

Since $PA+A^{T}P=-Q$ and $Ax_{f}=0$, Equation (16) can be written as follows:

(17) $$\dot{V}\left(x\right)=-\frac{1}{2}x^{T}Qx+\sum_{i=1}^{m}{\left(x-x_{f}\right)}^{T}PB_{i}u_{i}\left(t\right)x.$$

Substituting Equation (15) into Equation (17),

(18) $$\dot{V}\left(x\right)=-\frac{1}{2}x^{T}Qx-\sum_{i=1}^{m}\frac{1}{r_{i}}{\left[{\left(x-x_{f}\right)}^{T}PB_{i}x\right]}^{2}\leq 0.$$

According to Lyapunov stability theorem, the control law of Equation (15) guarantees the stability of system (12).

(ii) Optimality

The Hamiltonian function of the system is as follows:

(19) $$H=L\left(x,u\right)+V_{x}\left(x\right)\left(\left(A+\sum_{i=1}^{m}B_{i}u_{i}\left(t\right)\right)x\right),$$

where $L\left(x,u\right)=\frac{1}{2}x^{T}Qx+\frac{1}{2}\sum_{i=1}^{m}\frac{1}{r_{i}}{\left[{\left(x-x_{f}\right)}^{T}PB_{i}x\right]}^{2}+\frac{1}{2}u^{T}Ru$, $V_{x}={\left(x-x_{f}\right)}^{T}P$. The sufficient condition for optimality is

(20) $$\underset{u\in R^{m}}{min}\left[H\left(x,u\right)\right]=0.$$

From (20), we have

(21) $$\frac{\partial H}{\partial u_{i}}=r_{i}u_{i}+{\left(x-x_{f}\right)}^{T}PB_{i}x.$$

Replacing $u_{i}$ in (21) with (15) gives $\frac{\partial H}{\partial u_{i}}=0$, and replacing (20) with (15) yields

(22) $$\begin{array}{cc} H\left(x,u^{*}\right) & =\frac{1}{2}x^{T}Qx+\frac{1}{2}\sum_{i=1}^{m}\frac{1}{r_{i}}{\left[{\left(x-x_{f}\right)}^{T}PB_{i}x\right]}^{2}+\frac{1}{2}\sum_{i=1}^{m}\frac{1}{r_{i}}{\left[{\left(x-x_{f}\right)}^{T}PB_{i}x\right]}^{2}\\ & +\frac{1}{2}x^{T}\left(PA+A^{T}P\right)x-x_{f}^{T}PAx-\sum_{i=1}^{m}\frac{1}{r_{i}}{\left[{\left(x-x_{f}\right)}^{T}PB_{i}x\right]}^{2}\\ & =\frac{1}{2}x^{T}Qx-\frac{1}{2}x^{T}Qx\\ & =0. \end{array}$$

Therefore, the control law (15) is the optimal control law, which can minimize the performance index (13). Theorem 1 is proved. □

#### 3.2.2. Optimal Control of a Spin-1/2 Particle System

The spin-1/2 particle system is represented by Equation (3), where the initial state |ψ(0)〉=|0〉, and the target state $|\psi_{f}\rangle =|1\rangle$. Let $|\psi \rangle ={\left[x_{1}+ix_{3},x_{2}+ix_{4}\right]}^{T}$ to obtain the equation of state for the real vector *x*:

(23) $$\dot{x}=\left(A+B_{1}u_{1}\left(t\right)+B_{2}u_{2}\left(t\right)\right)x,$$

where $A=\left[\begin{array}{cccc} 0 & 0 & 2 & 0\\ 0 & 0 & 0 & 0\\ -2 & 0 & 0 & 0\\ 0 & 0 & 0 & 0 \end{array}\right]$, $B_{1}=\left[\begin{array}{cccc} 0 & 0 & 0 & 1\\ 0 & 0 & 1 & 0\\ 0 & -1 & 0 & 0\\ -1 & 0 & 0 & 0 \end{array}\right]$, $B_{2}=\left[\begin{array}{cccc} 0 & -1 & 0 & 0\\ 1 & 0 & 0 & 0\\ 0 & 0 & 0 & -1\\ 0 & 0 & 1 & 0 \end{array}\right]$,

$x_{0}={\left[1,0,0,0\right]}^{T}$, and $x_{f}={\left[0,1,0,0\right]}^{T}$. According to Theorem 1, the performance index is selected as $J=\frac{1}{2}\int_{0}^{\infty }\sum_{i=1}^{m}\frac{1}{r_{i}}{\left[{\left(x-x_{f}\right)}^{T}PB_{i}x\right]}^{2}+r_{1}u_{1}^{2}+r_{2}u_{2}^{2}dt$. Then, *Q* is a zero matrix, *P* is a matrix of identity that satisfies $PA+A^{T}P=-Q$, and the resulting optimal control law is $u_{i}^{*}=-\frac{1}{r_{i}}{\left(x-x_{f}\right)}^{T}B_{i}x\left(i=1,2\right)$. Next, take two examples and observe the evolution of $u_{i}^{*}$ corresponding to this case.

**Figure 9:**
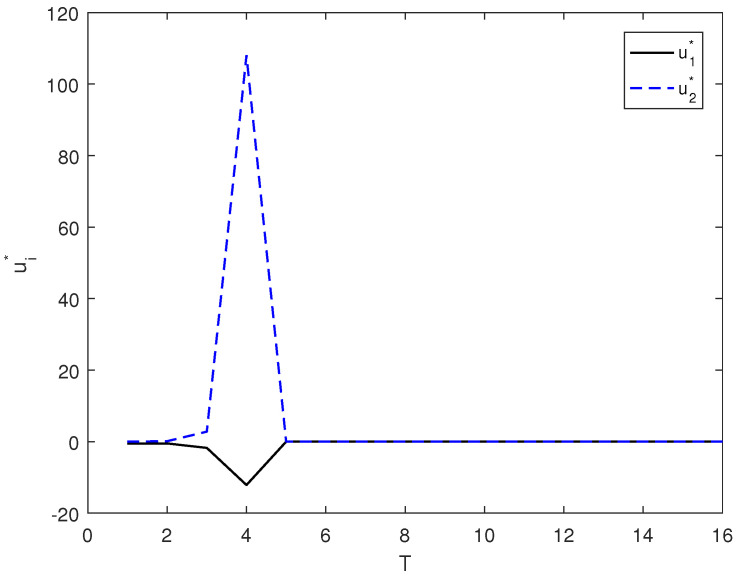
$u_{i}^{*}$ corresponds to the evolution process under $r_{1}=2$ and $r_{2}=1$.

**Figure 10:**
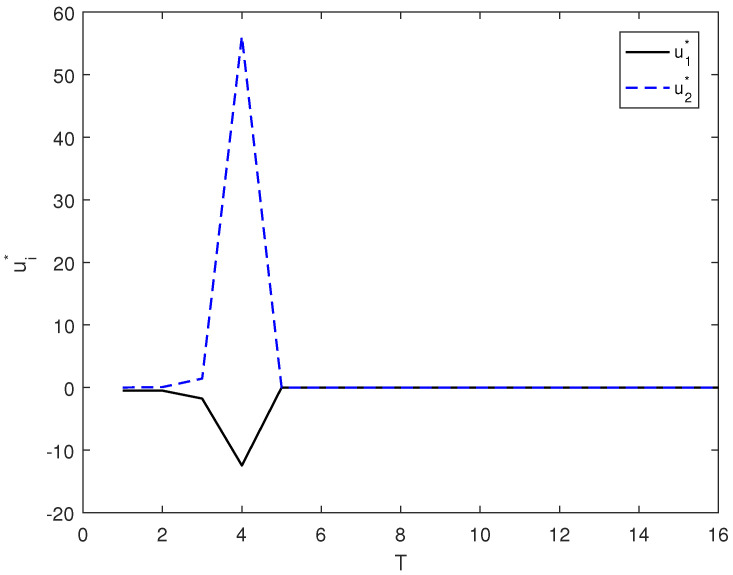
$u_{i}^{*}$ corresponds to the evolution process under $r_{1}=2$ and $r_{2}=2$.

(i) First example:

Taking $r_{1}=2$ and $r_{2}=1$, in Figure 9, we give the evolution of $u_{i}^{*}$ corresponding to this case.

(ii) Second example:

Taking $r_{1}=2$ and $r_{2}=2$, in Figure 10, we give the evolution of $u_{i}^{*}$ corresponding to this case.

The control law (7) is shown in Figure 7, and the optimal control of (15) is shown in Figure 9 and Figure 10. By comparing Figure 7, Figure 8, Figure 9 and Figure 10, it can be seen from the figure that with the optimal control of (15), system state can reach the target state faster.

## 4. Conclusions

This paper explores a quantum control design strategy that utilizes the Lyapunov function and the optimal control law for the quantum control system (3). We propose two distinct control law design methods. Numerical simulations were conducted for 3D and 5D systems, as well as for the spin particle system. The results demonstrate that the proposed control laws are effective. Notably, the first control law can stabilize the quantum system, an achievement not possible with a 3D system. Additionally, our control law is more straightforward than the one presented in [19].

## Data Availability

Data are contained within the article.

## References

[1] Timothy H.B. (2018). Blackbody radiation in classical physics: A historical perspective. Am. J. Phys..

[2] Don S.L., William R.S. (2024). Adiabatic invariance and its application to Wien’s complete displacement law of blackbody radiation. Am. J. Phys..

[3] Gkourmpis A. (2023). A demonstration of the photoelectric effect with sunlight. Phys. Teach..

[4] Lou C.G., Dai J.L., Wang Y.X., Zhang Y., Li Y.F., Liu X.L., Ma Y.F. (2023). Highly sensitive light-induced thermoelastic spectroscopy oxygen sensor with co-coupling photoelectric and thermoelastic effect of quartz tuning fork. Photoacoustics.

[5] Hsieh S.S., Katsuyuki T. (2024). Spectral information content of Compton scattering events in silicon photon counting detectors. Med. Phys..

[6] Bornikov K.A., Volobuev I.P., Popov Y.V. (2023). Notes on Inverse Compton Scattering. Mosc. Univ. Phys. Bull..

[7] Ikuta R. (2022). Wave-particle duality of light appearing in an intensity interferometric scenario. Opt. Express.

[8] Elinor Z.H., Yonatan D. (2023). Signature of Quantum Coherence in the Exciton Energy Pathways of the LH2 Photosynthetic Complex. ACS Omega.

[9] Daniel L., Martin H.G. (2016). Making and breaking bonds with absolutely localized molecular orbitals: An energy-decomposition analysis for bonded interactions. Abstr. Pap. Am. Chem. Soc..

[10] Peirce A., Dahleh M., Rabitz H. (1988). Optimal control of quantum-mechanical systems: Existence, numerical approximation, and applications. Phys. Rev. A Gen. Phys..

[11] Albertini F., D’Alessandro D. (2013). Control of a two-level quantum system in a coherent feedback scheme. Phys. A Math. Theor..

[12] Stefanatos D., Paspalakis E. (2020). A shortcut tour of quantum control methods for modern quantum technologies. Europhys. Lett..

[13] Koch C.P., Boscain U., Calarco T., Dirr G., Filipp S., Glaser S.J., Kosloff R., Montangero S., Schulte-Herbrüggen T., Sugny D. (2022). Quantum optimal control in quantum technologies. Strategic report on current status, visions and goals for research in Europe. EPJ Quantum Technol..

[14] Khaneja N., Brockett R., Glaser S.J. (2001). Time optimal control in spin systems. Phys. Rev. A.

[15] Cong S. (2014). Control of Quantum Systems: Theory and Methods.

[16] Kuang S., Cong S. (2008). Lyapunov control methods of closed quantum systems. Automatica.

[17] Wang X., Schirmer S. (2010). Analysis of Lyapunov method for control of quantum states. IEEE Trans. Autom. Control.

[18] Grivopoulos S., Bamieh B. Lyapunov-based control of quantum systems. Proceedings of the 42nd IEEE Conference on Decision and Control.

[19] Coron J.M., Grigoriu A., Lefter C., Turinici G. (2009). Quantum control design by Lyapunov trajectory tracking for dipole and polarizability coupling. New J. Phys..

[20] Bose S.S.C., Alfurhood B.S., Flammini F., Natarajan R., Jaya S.S. (2023). Decision Fault Tree Learning and Differential Lyapunov Optimal Control for Path Tracking. Entropy.

[21] Byoungsoo L., Walter J.G. (2012). Aeroassisted orbital maneuvering using Lyapunov optimal feedback control. J. Guid. Control Dyn..

[22] Rush D.R., Gordon G.P., Hanspeter S., John L.J. (2012). Lyapunov Optimal Saturated Control for Nonlinear Systems. J. Guid. Control Dyn..

[23] Jouili K., Madani A. (2023). Nonlinear Lyapunov Control of a Photovoltaic Water Pumping System. Energies.

[24] Yang Z., Yang J., Chao S., Zhao C., Peng R., Zhou L. (2022). Simultaneous ground-state cooling of identical mechanical oscillators by Lyapunov control. Opt. Express.

[25] Kuang S., Guan X.K. (2020). Robustness of continuous non-smooth finite-time Lyapunov control for two-level quantum systems. IET Control Theory Appl..

[26] Hou S.C., Yi X.X. (2020). Quantum Lyapunov control with machine learning. Quantum Inf. Process..

[27] Guan X.K., Kuang S., Lu X.J., Yan J.Z. (2020). Lyapunov Control of High-Dimensional Closed Quantum Systems Based on Particle Swarm Optimization. IEEE Access.

[28] Aleksandrov A., Efimov D., Dashkovskiy S. (2022). On input to state stability Lyapunov functions for mechanical systems. Int. J. Robust Nonlinear Control.

[29] Mirrahimi M., Rouchon P., Turinici G. (2005). Lyapunov control of bilinear Schrödinger equations. Automatica.

[30] Yu G.H., Yang H.L. (2024). Quantum control based on three forms of Lyapunov functions. Chin. Phys. B.

[31] Benallou A., Mellichamp D.A., Seborg D.E. (1988). Optimal stabilizing controllers for bilinear systems. Int. J. Control.

[32] Zhang Y.Y., Cong S. (2008). Optimal quantum control based on Lyapunov stability theorem. J. Univ. Sci. Technol. China.

[33] Franco B., Stefano M. (2019). Set-Theoretic Methods in Control.

